# Utilizing yeast chemogenomic profiles for the prediction of pharmacogenomic associations in humans

**DOI:** 10.1038/srep23703

**Published:** 2016-03-30

**Authors:** Yael Silberberg, Martin Kupiec, Roded Sharan

**Affiliations:** 1 Department of Molecular Microbiology and Biotechnology, Tel-Aviv University, Tel Aviv, Israel; 2 The Blavatnik School of Computer Science, Tel-Aviv University, Tel-Aviv, Israel

## Abstract

Understanding the genetic basis underlying individual responses to drug treatment is a fundamental task with implications to drug development and administration. Pharmacogenomics is the study of the genes that affect drug response. The study of pharmacogenomic associations between a drug and a gene that influences the interindividual drug response, which is only beginning, holds much promise and potential. Although relatively few pharmacogenomic associations between drugs and specific genes were mapped in humans, large systematic screens have been carried out in the yeast *Saccharomyces cerevisiae*, motivating the constructing of a projection method. We devised a novel approach for the prediction of pharmacogenomic associations in humans using genome-scale chemogenomic data from yeast. We validated our method using both cross-validation and comparison to known drug-gene associations extracted from multiple data sources, attaining high AUC scores. We show that our method outperforms a previous technique, as well as a similar method based on known human associations. Last, we analyze the predictions and demonstrate their biological relevance to understanding drug response.

In the genomic era, personalized medicine has been in the focus of the medical field with the promise of safer and more efficient patient-tailored treatment^1^. To realize it, one must gain a deeper understanding of individual drug responses and the underlying genetic variance involved in their activation. Pharmacogenomics (PGx) is the study of the genetic basis underlying individual variation in drug response. As such, identifying PGx associations between genes that affect drug response and the corresponding drugs is the first step toward patient- tailored medicine. By its nature, PGx associations are not constrained to physical drug-gene interaction, and may involve genes that indirectly affect drug response such as disease-related genes^2^, in addition to physical interactions such as drug receptors or metabolizing enzymes^3^. The number of PGx associations started to bloom with the emergence of high throughput genomic technologies; however, the discovery of PGx associations is still far from completion^4^,^5^,^6^ with only a few thousands associations available in public databases such as PharmGKB^7^ and GWASCatalog^8^.

A few methods for the automatic prediction of PGx associations have been proposed in the literature. Hansen and co-workers^9^ characterized a gene using its network neighbors and scored its association to a drug, based on known associations of the gene neighbors to similar drugs. Their method thus crucially depends on the availability of protein-protein interaction data and prior knowledge on PGx associations. Another study^10^ used biomedical knowledge and functional annotations to predict genes that either affect drug response or are involved in disease. The predictions of this method, however, lack the distinction between the drug-related genes and the disease-related genes, and provide only a list of putative genes without the relevant context (i.e. its associated drugs or diseases). A genome-scale, unbiased effort for PGx discovery was performed in the context of cancer research: Barretina and co-workers evaluated extensive genomic data from different cancer cell lines and analyzed variations in cell lines responses to compounds in order to recover key genomic predictors of drug sensitivity^11^. However, these predictions were made in the context of a specific disease.

Earlier work known as the connectivity map project^12^ set to map genes that are involved in drug response in humans, with the goal of finding novel connections between diseases and drugs. Gene expression response was measured for over a thousand drugs in multiple cell lines, identifying genes whose activity is altered upon drug administration. While this is a valuable resource about the workings of drugs, the resulting data cannot be directly used to infer pharmacogenes, i.e., genes that affect drug response, but rather identifies the effect of a drug by highlighting genes which alter their activity in its presence.

Remarkably, genes that affect the cell’s sensitivity to drug administration have been carefully cataloged in yeast for over a decade. A collection of strains is available, each deleted for a different gene. The complete deletion library in yeast was screened in the presence of a large number of chemical compounds, and the growth phenotype of all strains were measured^13^. Thus, the so-called chemogenomic profile of a drug captures knockout strains whose sensitivity to the drug is altered compared to the wild type. Two main types of profiles exist: (i) The haploinsufficient profile (HIP), which measures the growth fitness (i.e., the growth ability of a knockout strain vs. the wild type) of a diploid yeast strain heterozygous for the gene deletion in the presence of a drug, and (ii) the homozygote profile (HOP), which quantifies the fitness of a diploid strain homozygote for a particular gene deletion; this analysis is, of course, applicable for non-essential genes only. Previous studies have demonstrated the relevance of these data in uncovering drug mechanism of action^14^, drug targets^15^,^13^ and drug off-targets^16^ in yeast. For example, Parsons and co-workers^10^ screened 12 inhibitory compounds and clustered the resulting chemogenomic profiles with a set of genetic interaction profiles to illuminate the drug’s targets and drug’s pathways in yeast. They showed that the chemogenomic profile of an inhibitory drug resembles the genetic interaction profile of the gene the drug inhibits, allowing the discovery of drug targets and proteins related to a drug’s mode of action. Their method is, however, limited to inhibitory compounds, and provides prediction to drug-target genes in yeast only. Ericson *et al.*^16^ aimed at identifying off target effects of psychoactive drugs in yeast by screening 81 bioactive drugs and analyzing the resulting chemogenomic profiles. Their analysis again remains bounded to yeast genes and pathways only.

The projection of the chemogenomic association in yeast to infer valuable drug mode-of-action in humans is a complex task, which requires careful identification of relevant chemogenomic associations, as well as the development of a method allowing the projection of the identified drugs and yeast genes to human genes and relevant drugs of interest.

In this paper, we utilize three of the largest chemogenomic experiments available^17^,^18^,^19^ to predict pharmacogenomic associations in humans based on the similarity between human and yeast genes, drug similarity measurements and the measured effect of a drug on a yeast deletion strain. In cross-validation our method obtains a very high area under the receiver operating characteristic curve (AUC) of 0.95, outperforming previous methods based on existing knowledge of human drug-gene associations. Our predictions are also in accordance with well established drug-gene association data sources that were not used in the learning stage. Genes involved in the predicted PGx associations were found to be enriched with biologically relevant processes.

## Results

**Figure 1:**
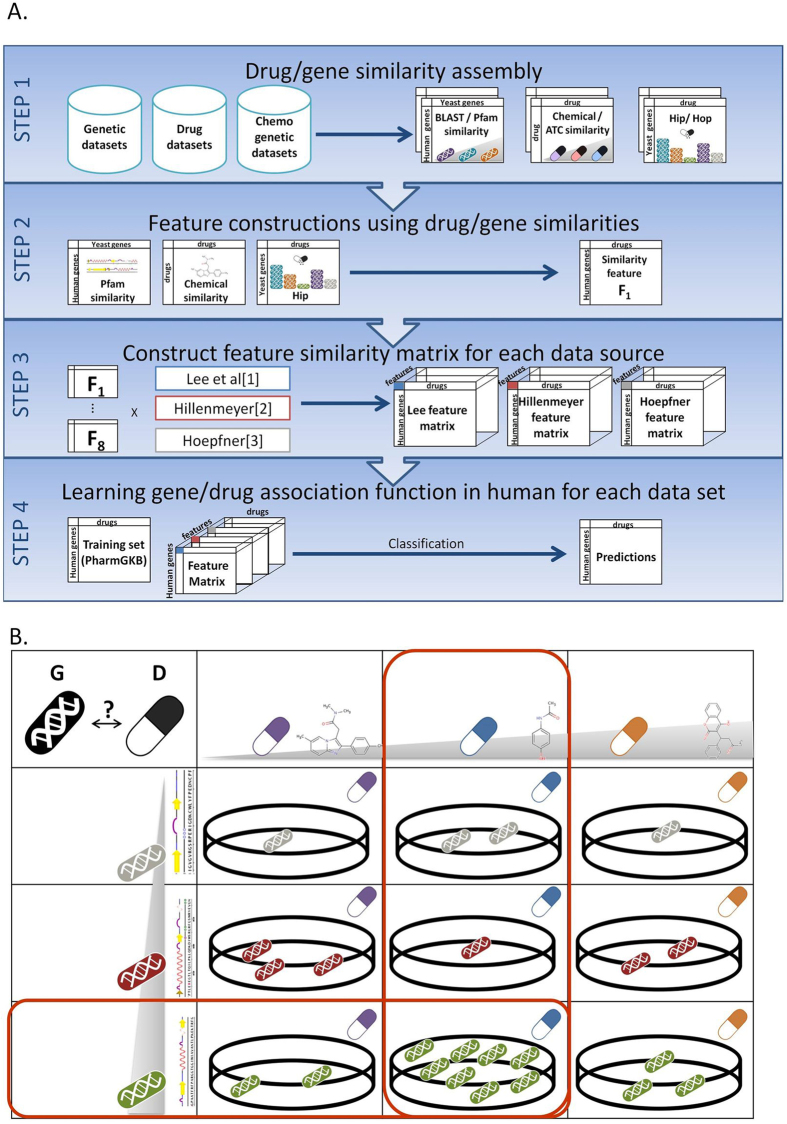
(**A**) Algorithmic pipeline: Step 1: using genetic datasets (e.g. PFam), drug datasets (e.g. DrugBank) and chemogenetic datasets (e.g. Lee) to construct gene similarity measurements, drug similarity measurements and HIP/HOP scores respectively. Step 2: Example of how to combine the three similarity measurements (gene similarity, drug similarity and HIP/HOP score) to construct one feature score. Step 3: Generating eight feature scores for each of the three main HIP/HOP data-sources, resulting in three three-dimentional feature matrices. Step 4: Uniting the three feature matrices into one matrix with 24 features. Using true PGx association extracted from PharmGKB as the positive training set, and applying Random Forest classifier to predict PGx associations. (**B**) Feature construction example, demonstrating step 2 in the algorithmic pipeline. The feature score for a given PGx association between a drug D, and human gene G, is the maximal geometric mean of three measurements, across all drugs and genes in a chemogenomic database. In this example the maximal score (marked in red) is achieved by the geometric means of the three following measurements: (i) the chemical similarity between the query drug and the drug marked in blue (ii) the domain similarity between the human query gene and the yeast gene marked in green and (iii) the HIP chemogenomic association between the drug marked in blue and the yeast knock-out gene marked in green.

We designed a novel prediction scheme that scores a potential PGx association by its similarity to a chemogenomic interaction in yeast and the confidence of that interaction (Fig. 1). Each potential PGx association was scored by applying a machine learning technique on a set of eight unique features, each representing a distance to a chemogenomic association based on three similarity measurements. The core of our method is the feature score, which takes into account the similarity between the query drug and all drugs tested in yeast, the similarity between the human and yeast genes, and the chemogenomic score as observed in yeast. The feature score was calculated by applying the geometric mean on the three measurements, across all drugs and yeast genes in a chemogenomic database, and then choosing the maximal score (Fig. 1B). The maximal score represents the most similar drug-gene pair in yeast, with an observable chemogenomic reaction, eliminating the noise raised from genes that did not alter drug response in yeast, and the noise raised from a chemogenomic association for non-similar drugs or non-similar genes to the query pair. The geometric mean assigns to each of the tree measurements an equal contribution to the final feature score. Formally, a feature score for PGx association between a drug D and a human gene G is calculated as follow:


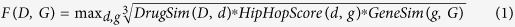


where *d, g* range over all drugs and yeast genes present in the relevant chemogenomic data set.

We constructed two drug-drug similarity measures (chemical- and ATC- based), two gene-gene similarity measures (sequence- and domain-based) and two types of chemogenomic association scores (HIP and HOP) (see Methods for a full description). Combining three out of the six similarity measurements into a single score results in a set of eight features per potential association (Table 1).

**Figure 2:**
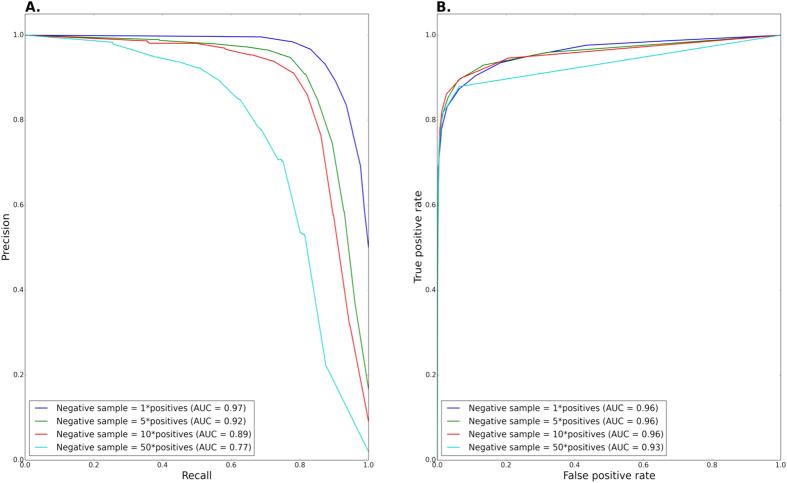
Cross validation. (**A**) Precision recall graph evaluating cross validation performance, using different sizes of negative sets (**B**). ROC graph evaluating cross validation performance, using different sizes of negative sets.

We obtained three large scale data sources of chemogenomic interactions from Hillenmeyer and co-workers^17^, Lee and Co-workers and Hoepfner and co-workers^19^, each of which contains both HIP and HOP scores for a different set of drugs tested (Methods). From each data source we extracted the HIP/HOP scores provided by the author describing yeast chemogenomic profile of drug administration. We considered only drugs and genes which were mapped to our ground set, to which drug and gene similarity measurements were available, and thus feature construction was feasible. We then used the three data sources to derive 24 features, eight features derived from each data set, characterizing each potential PGx association. Finally, we trained a Random Forest classifier on the set of 24 features to predict PGx associations. The process resulted in predictions of PGx associations between 27,311 human proteins, to which gene similarity calculation was feasible, and 1,333 drugs to which drug similarity calculation were feasible, covering the majority of FDA approved drugs. All our reported results are with respect to this *ground set* of genes and drugs. We evaluated our results using a 10-fold cross validation against gold standard PGx associations retrieved from PharmGKB (Methods), consisting of 680 direct drug-gene associations and 760 additional associations extrapolated from drug-gene class relations. Both types of associations gave similar performance in cross validation (AUC of 0.92 ± 0.007 for the direct associations vs. 0.95 ± 0.004 for the complete set; area under the precision-recall curve (AUPR) of 0.93 ± 0.006 vs. 0.96 ± 0.003, respectively), hence we used the entire set of 1,440 associations as our gold standard in the sequel. We repeated the cross-validation with different sizes of negative sets ranging from a negative set whose size is equal to the positive set and up to 50-fold larger. The resulting AUCs and areas under the precision-recall curves are summarized in Fig. 2. As observed in the figure, while the AUCs are unaffected by class imbalance, the AUPRs deteriorate as the number of negative examples increases. However, even in the most unbalanced setting, we were able to obtain a high precision score of 0.98 (for a classification score cutoff of 1), although at a lower recall value of 0.25. Henceforth, we applied this strict cutoff in order to minimize false positive predictions.

To evaluate the contribution of the yeast chemogenomic interactions to the prediction power of our method we applied our method on the same subset of PGx associations, omitting the chemogenomic interactions from feature calculation. To this end, we used a similar scheme that scores a feature for a potential PGx association by its similarity to known PGx associations in humans using drug and gene similarity measurements only (Methods). This method yielded an AUC score of 0.84, demonstrating the added value obtained by integrating yeast chemogenomic interaction information into the prediction framework. To validate the robustness of the results, we excluded 5% of the drugs with the highest sums of CGI scores from each data set (Methods) and repeated the feature calculation and classifier learning steps without this set of drugs. We verified that neither the quality of predictions (as measured in cross validation), nor the quantity of the predictions is affected by the drug removal. Indeed, both AUC and AUPR remain essentially unchanged (AUC = 0.96 ± 0.003 and AUPR = 0.96 ± 0.002), and the total number of predicted PGx associations remained similar with 136,840 ± 25,680 predictions in the new setting vs. 118,901 ± 16,912 in the original set (averaged over 10 random negative sets).

We further compared our method with the one previously published by Hansen and co-workers^9^, which is, to the best of our knowledge, the only previous method predicting PGx associations in a large scale. Hansen used two types of drug-gene associations, two measures of drug-drug similarity and the protein-protein interaction (PPI) network to construct a set of four features for a potential PGx association (Methods). Each feature represents the similarity of a query drug to a drug known to associate with a PPI-neighbor gene of the query genes, based on the assumption that neighbor genes tend to associate with similar drugs. Each PGx association is scored by applying a logistic regression classifier on the set of the four features (Methods). To compare our method with that of Hansen, we defined a *valid* association set for each method as the set of associations for which feature calculation was feasible, based on data availability (Methods). Out of the 3075 PGx associations retrieved from PharmGKB, 1400 (45%) were valid for our method, while only 389 (13%) associations were valid for Hansen’s method, demonstrating the more than 3-fold power of our method to detect true associations. Out of these associations, 140 were valid for both methods. We trained each method on the set of all its valid PGx associations (minus the intersection set) and an equally sized set of random negative associations, and compared their performance on the 140 common associations. Our method obtained an AUC of 0.95 vs. 0.74 when using Hansen’s method.

**Figure 3:**
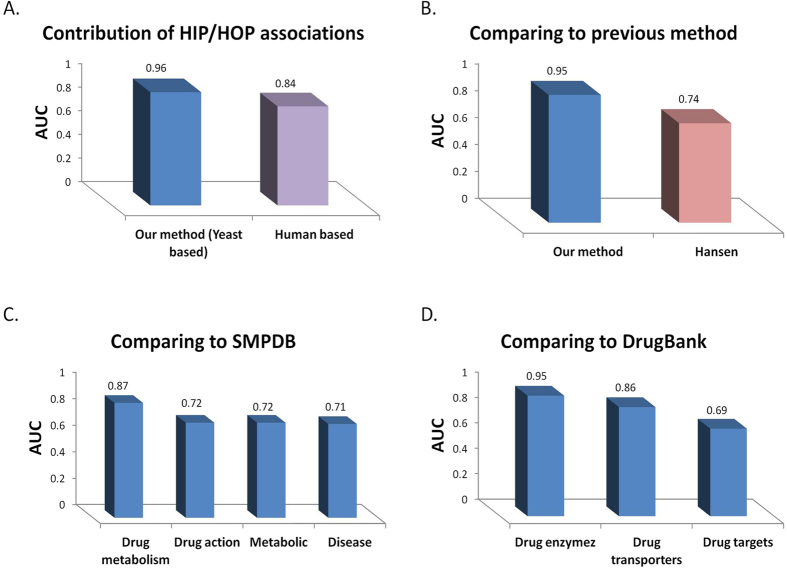
Performance evaluation. (**A**) Areas under the curves (AUC) obtained in a 10-fold cross-validation setting comparing our method (yeast based) to a similar human-based method, omitting the HIP/HOP score from feature construction. (**B**) AUC scores obtained for 140 joint valid associations in both our method and Hansen’s. (**C**) Performance evaluation on external data sources: A comparison of our predictions to different pathway categories downloaded from The Small Molecule Pathway Database (SMPDB). (**D**) A comparison of our prediction to different association types extracted from DrugBank.

Next, we validated our predictions against two external data sources: (i) Drug-gene associations downloaded from DrugBank^20^, a chemoinformatics data source of drugs and small molecules and their targets; and (ii) the Small Molecule Pathway Database (SMPDB)^21^, a manually curated database of small molecule pathways in humans. The results are summarized in Fig. 3.

For the first comparison, we used a set of drug-gene associations downloaded from DrugBank. Each drug stored in DrugBank may be associated with one or more proteins in each of the following categories: (i) Drug target: representing a protein to which the drug binds, leading to the therapeutic effect. (ii) Enzyme, catalyzing chemical reactions in which the drug serves as the substrate. (iii) Transporter: a membrane bound protein which transfers the drug across membranes, into or out of the cells. Or (iv) carrier: a protein facilitating the transmission of the drug to the transporter protein. We extracted 1,239 drug-target associations between 581 genes and 496 drugs from our ground set; 1,106 drug-transporter associations between 76 genes and 423 drugs from our ground set; And 2,562 drug-enzyme associations between 126 genes and 742 drugs from our ground set. Only two drug-carrier associations were available for our ground set, and thus they were dismissed from further analysis. Our predictions obtained AUC scores of 0.69, 0.86 and 0.95 with regard to drug targets, transporters and enzymes, respectively. This suggests that metabolic enzymes may have a higher effect on the drug response than drug targets, e.g. failing to metabolize a drug may cause severe side effects, which may be noticeable in cell viability in yeast^3^. Alternatively this observation may result from a higher conservation between human and yeast metabolic enzymes than drug-target genes, many of which lack a yeast homolog^16^, allowing for a more accurate prediction of metabolic enzymes using our method.

To compare our predictions to SMPDB, we downloaded 718 pathways for over 600 drugs, 244 of which were present in our ground set. For each of the six pathway categories stored in SMPDB (metabolic, drug metabolism, drug action, disease, signaling and physiological) we constructed a drug-gene association matrix based on pathways associated with that category and their corresponding genes and drugs. The association was made based on the assumption that genes involved in a drug’s pathway are likely to confer sensitivity to the drug when mutated. We generated ROC curves for each pathway type independently (Methods). The resulting AUC scores ranged between 0.71 and 0.87 for the different pathway types, with an average of 0.75 (Fig. 3). The highest AUC score belonged to the ‘drug metabolism’ category, in line with previous studies reporting that drug metabolism plays a key role in variation to drug response among individuals^3^.

**Figure 4:**
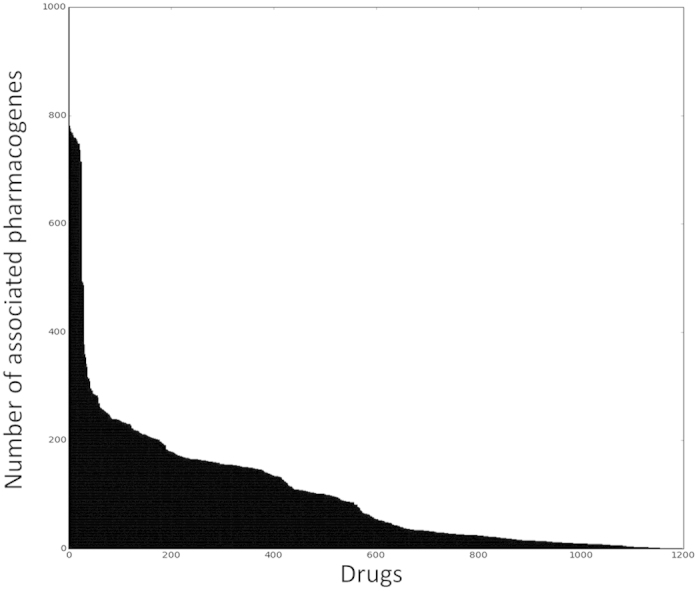
Distribution of number of pharmacogenes associations per drug.

After establishing the accuracy of our method, we turned to evaluate our predictions. By focusing on the top predictions with a perfect classification score of 1, we obtained 124,010 novel predictions between 3,586 proteins and 1,197 drugs (out of 36,405,563 possible associations between 27,311 proteins and 1,333 drugs). Top predictions are provided in supplementary Table S1, the full prediction list is available for download at www.cs.tau.ac.il/~roded/PGxPredictions.zip. Figure 4 displays the distribution of the number of PGx associations predicted per drug. While over 85% of the drugs were associated with less than 200 pharmacogenes, we found a small group of 26 drugs associated with over 600 genes (see Fig. 4). Out of the 26 drugs, 23 belong to the antidepressant class (hyper-geometric p-value <2.5e^−26^, see Table 2). We noticed that these drugs also have significantly more known PGx associations stored in PharmGKB than other drugs (average of 8.65 vs. 0.93, Wilcoxon ranksum p-value <1.6e^−12^) and significantly more known associations with enzymes stored in DrugBank (average of 3.9 vs. 2, p-value <1.2e^−4^). To evaluate the relevance of the predicted pharmacogenes to antidepressant treatment, we compared the list of the predicted pharmacogenes to a set of proteins known to associate with depression, retrieved from Diseases^22^ (Methods). Out of the 55 depression-related proteins retrieved from Diseases, 34 were found in our ground set, six of which were found in the predicted list of antidepressant-pharmacogenes (hypergeometric p-value < 1.2e^−6^).

To investigate the biological mechanism underlying the predicted response to antidepressants, we analyzed the functional annotations of the corresponding pharmacogenes. Overall, 112 GO processes were enriched in this set of pharmacogenes (e-val < 0.01). The top three enriched annotations involved drug metabolism (xenobiotic metabolic process, drug catabolic process and exogenous drug catabolic process, q-val < 3e^−20^). Indeed, variations in the activity of metabolic enzymes are currently the genetic factors most associated with response to antidepressants and antipsychotics^23^.Moreover, antidepressants are a class of drugs known to be ineffective in a particularly high percentage of patients in comparison to other drug classes (up to 50% of individuals)^24^,^25^,^26^. The high number of metabolic enzymes exhibiting sensitivity for antidepressant response may suggest one explanation for this high diversity in drug response among the population. More interesting perhaps, was the GO-category ‘response to hormone’, which was also enriched in this set of genes (q-value < 1.4e^−4^). Indeed, it is widely acknowledged that hormones, and especially sex hormones, play a role in depression mechanisms and affect the success of antidepressant treatment^27^,^28^,^29^,^30^. Within this GO-category we found four proteins belonging to the GNB family (GNB1, GNB3, GNB5 and GNB2L1) that are predicted to associate with this set of antidepressant drugs. Out of these proteins, GNB3 is a serotonin-related gene, whose polymorphism was reported to be associated with depression in a meta-analysis^31^. A following study associated polymorphisms in this gene with differences in short-term response to antidepressant treatment^32^.

At the other extreme we checked for drugs with the smallest number of associated genes and found 252 drugs (19%) that were associated with five or less pharmacogenes. These drugs were enriched with several categories including ‘contrast medium’ (hyper-geometric p-value < 8.6e^−12^), ‘anti-inflammatory agents’ (hyper-geometric p-value < 7.4e^−10^) and ‘diagnostic agents’ (hyper-geometric p-value < 2e^−8^), see Table 3. Many of this categories (e.g. anti-inflammatory agents), are not expected to be active in yeast, explaining the low number of association predicted for this drugs. Among the drugs with a small number of predicted PGx, we found three anabolic agents: oxandrolone, nandrolone phenpropionate and ethylestrenol; all three were associated with the LATS2 protein (large tumor suppressor kinase 2), a serine/threonine kinase that regulates cell organization during mitosis. LATS2 is known to interact with Androgen Receptor, AR, which is the known target of both nandrolone phenpropionate and oxandrolone. Moreover LATS2 was shown to mediate repression of AR activity^33^. Nandrolone phenpropionate, which is used to treat, among others, breast carcinoma, was further associated with the MDC1 protein (mediator of DNA-damage checkpoint 1). The level of MDC1, a critical component in DNA damage response, was shown to be aberrantly reduced in 30% of breast carcinoma tissues^34^, and its expression was further suggested as a prognostic marker in early-stage breast cancer^35^. Last, MDC1 was shown to participate in AR-mediated p53-dependent apoptosis in prostate cancer^36^.

To test whether drugs with many PGx predictions affect the results, we repeated the cross-validation and external evaluation steps after removing drugs with more than 600 or 150 predicted associations (Methods). While removing drugs according to the higher cutoff did not affect the performance, removing drugs with over 150 predicted associations slightly deteriorated the performance for both cross validation and external validation steps, possibly due to the significantly lower number of positive example in the training set (1400 vs. 479). The results are summarized in Table 4, further demonstrating the robustness of our approach.

## Discussion

We have developed a novel method for the prediction of PGx associations, by harnessing chemogenomic data from yeast. Using our method we were able to obtain predictions of PGx associations between 27,311 human proteins and 1,333 drugs (covering the majority of FDA approved drugs), based on a small subset of relevant drugs screened in yeast. Our method was cross validated, as well as validated against independent test data, yielding high AUC scores. We demonstrated the added value of using chemogenomic associations from yeast by comparing to a similar method based on existing knowledge in human.

With the rapid growth of current pharmacogenomic knowledge, the predictive power of our method is expected to increase. In addition, our method can easily be extended by introducing new similarity measurements, expected to improve the accuracy of the prediction. For example, the gene similarity measurements used here (BLAST and PFam based), are both structure-based. While Blast similarities were limited to significant alignments only, retrieving a small set of human homologs for each yeast gene, the PFam similarity measurement is able to match more human orthologs per yeast gene, resulting in more potential PGx associations. The different measurements are balanced by the classifier, which weights the different features according to their contribution in identifying true associations. Expanding the scope of gene similarity measurements (e.g. by introducing GO-based similarity), may both increase the range of our prediction by finding additional similar human genes, and refine the current predictions by requiring genes to be similar in multiple aspects. In a similar manner, our method is readily extensible to other sources on chemogenomic associations. Chemogenomic association derived from *Schizosaccharomyces pombe*^37^, *Candida albicans*^38^ or *Escherichia coli*^39^ may not only refine our prediction, but also expand the collection of potential human orthologs. Last, using data sources such as chemogenomic studies based on genes over expression^40^ or multi-copy variant^41^ may lead to better prediction power, by providing a more comprehensive description of cell responses to drug administration.

## Methods

### Data Sets

The following data was extracted from DrugBank^20^: Anatomical Therapeutic Chemical (ATC) classification and canonical Simplified Molecular Input Line Entry Specification (SMILES) were extracted and used for drug similarity measurements. Out of 7,740 drugs in DrugBank, 1,333 drugs with both chemical structure (SMILES formulation) and ATC classification were available, enabling drug similarity calculation. All our reported results are with respect to this *ground set* of drugs. Additionally drug transporters, drug enzymes and drug targets were obtained and used for external validation.

Chemogenomic interactions (HIP/HOP scores) were obtained from Hillenmeyer and co-workers^17^, Lee and co-workers^18^ and Hoepfner and co-workers^19^, henceforth Hillenmeyer, Lee and Hoepfner, respectively. For each data set, scores provided by the authors which best describe gene sensitivity to the relevant drug were used as follows: for Hillenmeyer, we extracted the fold-change of a knock-out strain in the presence of drug vs. the wild type. This score was measured as a simple log_2_ ratio of the mean control intensity vs. treatment intensity. Scores were available for the sensitivities of 4,769 and 5,337 genes in 418 and 726 redundant conditions for the homozygote (HOP) and heterozygote (HIP) set, respectively. From this data set 22 HOP conditions and 60 HIP conditions were mapped to drugs from the ground set using generic drug names resulting in a set of 104,918 chemogenomic associations used in the HOP dataset and 320,220 HIP associations. Hoepfner supplied the MAD logarithmic (MADL) scores of each strain with respect to the average of the control sample. Scores were retrieved for 1,776 drugs and for 4,913 and 4,845 genes from the HIP and HOP datasets, respectively. Out of the 1,776 drugs, 18 were mapped to the ground set, creating a set of 88,434 HIP associations and 87,210 HOP associations. Last, the fitness defect score (FD-score) was extracted for Lee. This score represents the log_2_ ratio between the signal from the control samples to that from the chemical sample was. The FD-Score matrix contains information about 3,250 compounds, 4,810 HOP and 1,095 HIP genes. From these compounds, 111 drugs were mapped to the ground set, creating a set of 533,910 HOP associations and 121,545 HIP associations. In the case of multiple experiments for the same drug, the experiment with the highest drug concentration was chosen to represent the drug effect (26 drugs from Lee and 17 and 45 drugs from the Hillenmeyer HOP and HIP data sets respectively). To enable data integration we normalized all scores to be in the range [0, 1] by dividing the absolute value of HIP/HOP scores by the maximal score in the data set. The normalized scores approximate the strength of the effect that a gene deletion has on a cellular reaction to a drug.

For gold standard PGx associations, a set of 3,490 manually curated studies was downloaded from PharmGKB^7^, each study reporting associations between genes and drugs or drug classes. Overall, these records spanned 33 unique drug classes, with an average size of 12.2 drugs. They gave rise to 4,434 drug-gene associations and additionally 5,687 associations obtained by extrapolating gene-drug class associations to their corresponding gene-drug associations. Constraining the associations to the ground-set yielded 1,440 gold standard associations between 362 drugs and 203 genes, 680 of which (between 191 drugs and 188 genes) derived directly from gene-drug association records without extrapolation.

For gene similarity measurements we downloaded: (i) BLAST sequence similarity between 3,711 yeast genes and 9,621 human genes from the Saccharomyces Genome Database (SGD)^42^, and (ii) PFam domains files for yeast (tax id = 559292) and human (tax id = 9606) from PFam version 27.0^43^. The yeast domain file contained domain information for 4,908 yeast proteins, and the human domain file contained domain information for 51,444 human proteins.

For validation, drug pathways were downloaded from the ‘small molecule pathway database’ (SMPDB) version 2.0^21^.

Finally, to reproduce Hansen method, we downloaded 350,029 human protein-protein interactions spanning 12,561 proteins from InWeb^44^.

### Similarity Measures

We computed two drug-drug similarities and two gene-gene similarities. All similarities were normalized to be in the range [0, 1]. The drug similarities we used are:

1. Chemical similarity: chemical similarity was defined as the Tanimoto score between the chemical fingerprints of two compounds, i.e. the size of intersection over union of chemical elements present in the chemical fingerprints of the compounds. The hashed daylight-like fingerprint was computed for each compound from its SMILES structure using the open source Python library RDKit with default parameters.
2. ATC based similarity: World Health Organization (WHO) ATC classification system^45^ is a five-level hierarchical classification system that categorizes drugs according to the anatomical group on which they act, their therapeutic effect, and their chemical characteristics. We used the top three levels, omitting chemical characteristics to create the ATC similarity measure. The similarity score was defined as the lowest common ancestor ATC level in the hierarchical tree across all ATC categories assigned to the drug.

For gene-gene similarities we used:

1. Sequence similarity: The similarity between yeast and human proteins was defined as the percentage of the yeast protein aligned to the human protein, considering only significant alignments (E-value < 0.01). In the case of multiple alignments between two proteins, the largest aligned portion was used.
2. Domain similarity: We defined the similarity between a pair of proteins as the Jaccard score between their corresponding domains after filtering for domains present in both species.

For each similarity measurement, all similar genes/drugs were considered, creating many-to-many mapping. This type of mapping allows for consideration of all similar entities in feature calculation. E.g., by using many to many mapping in gene similarity we allow not only the closest homolog to affect feature score, but rather all homologs are consider and the chemogenomic association obtaining maximal score across all three similarity measurement is used.

All similarity measurements are provided in Supplementary Tables S2, S3,S4, S5.

### Classification methodology

We constructed classification features by combining drug-drug similarity, human-yeast gene similarity and drug-gene association measurement (i.e. yeast chemogenomic association scores). Each feature is a combination of one of the two drug similarity measures, one of the two gene-similarity measures, and one of the two chemogenomic association scores (either HIP or HOP), resulting in a set of eight features describing each query PGx association, obtained from each data set.

We trained a Random Forest classifier on the set of 24 features obtained from the three data sets. Random forest was implemented using Python scikit-learn library with default variables. Predict_proba functions was used to calculate the predicted class probability score which represents the mean predicted class probability of the trees in the forest, while the probability returned by a single tree is the normalized class probability of the leaf a sample lands in. To predict novel PGx associations we used the full training set from PharmGKB including 1,440 true associations and an equally sized randomly chosen negative set. Negative associations were generated in random out of 36,404,123 drug-gene pairs not known to associate.

### Performance evaluation

To evaluate the accuracy of our prediction method, we performed 10 independent runs of 10-fold cross-validation, choosing in each run a different set of randomly chosen negative examples (non-associations). The training set includes 1,440 true drug-gene PGx associations extracted from PharmGKB between 362 drugs and 203 human genes and a randomly generated set of 1,440 associations, not included in the positive set, simulating non-PGx associations. Each cross-validation run consists of ten iterations, in each we hide 10% of the associations, train on the remaining associations and test our performance on the hidden set. We score the prediction performance using the area under the receiver operating characteristic curve (AUC) and the area under the precision-recall curve (AUPR). We repeated the cross-validation process with different sizes of negative sets, ranging from the same size as the positive as to 50-fold higher.

To test whether the results are biased by a possibly high conservation between yeast genes and known human PGx genes, we repeated the test with a set of negative genes with the same conservation level as the genes in the positive set. To this end, we first obtained for each human gene the matching yeast gene with the highest BLAST score, retaining only significant matches. We then binned the scores into 50 groups according to the percentage of the yeast protein aligned to the human protein. From each bin we chose a random set of human genes to serve as negative examples in a frequency proportional to the representation of this bin in the positive set. Repeating the 10-fold cross validation test ten times with different random sets of negative associations, yielded similar AUCs (mean AUC = 0.95 ± 0.003) and AUPRs (mean AUPR = 0.97 ± 0.002) as the original negative set.

To validate the robustness of the method, we removed from each yeast chemogenomic dataset the 5% drugs with the highest sums of normalized chemogenomic association scores. Overall one out of 18 drugs was removed from Hopfner Hip and Hop data set (total of two drugs), three out of 60 drugs were removed from Hillenmeyer Hip dataset, 2 out of 22 drugs were removed from Hillenmeyer Hop data set, and six out of 111 drugs were removed from Lee Hip and Hop data sets each (total of 12 drugs). The feature construction and learning steps were then repeated without these drugs.

Last, to evaluate the influence of drugs with multiple predictions on the performance of both drug prediction and subsequent evaluations, we removed drugs with over 600 or 150 PGx predictions from the training set and repeated the learning and subsequent analysis. The evaluation was done in two steps: (i) filtering drugs with over 600 PGx predictions resulted in a set of 1,307 drugs to which 1,215 positive example were available from PharmGKB. The set of drugs filtered for over 150 PGx predictions resulted in a set of 982 drugs to which 479 positive examples were available. Cross validation was done on these subsets of drugs, with the available positive examples and random set of negative examples of the same size as the positive set. We repeated the cross validation ten times with different random sets of negative examples and evaluated performance using AUC and AUPR scores. (ii) Obtaining the full set of PGx predictions for each set of drugs. Prediction scores were compared with gold-standard drug-targets associations, drug-enzyme associations and drug-transported associations obtained from DrugBank for the corresponding set of drugs. We evaluated the performance using AUC scores for each association type. The filtered set of drugs yielded 2,485, 1,011 and 1,215 gold- standard associations for drug-enzymes, transporters and targets respectively for the set of drugs filtered for maximal 600 PGx predictions and 1,588, 771 and 886 associations respectively for drugs with less than 150 predicted PGx associations.

### Constructing SMPDB association matrices

We iterated over all pathways downloaded from SMPDB and matched all proteins associated with a pathway to all drugs associated with the same pathway. This resulted in a set of both physical and indirect associations between a drug and a set of genes associated with its pathway. Next we constructed a drug-gene association matrix for each of the six pathway categories based on associations extracted for all pathways cataloged into that category. Matrices representing ‘Physiological’ and ‘Signaling’ categories which spanned only 3 and 4 drugs from the ground set, respectively, were discarded from further analysis. We compared the classifier score to each of the remaining four category types to compute a ROC curve.

GO enrichment analysis was carried out using the Gorilla online analysis tool^46^.

To compare the predicted antidepressant pharmacogenes to a list of depression-related genes, we’ve downloaded a set disease-gene association from Diseases database^22^. We extracted genes associated with disease names containing ‘depression’ or ‘depressive’ keywords from the filtered text-mining table. The following diseases were retrieved: ‘Postpartum depression’, ‘Major depressive disorder’, ‘Atypical depressive disorder’ and ‘Endogenous depression’. A total of 55 unique genes were associated with this set of diseases, 34 of which were mapped to our ground set. From the set of 733 pharmacogenes associated with antidepressants, 233 were mapped to generic gene names allowing their comparison to the genes extracted from Diseases. We applied accumulative hypergeometirc test on the intersection protein to obtain the p-value.

### Comparison to previous methods

To compare our method to that of Hansen and co-workers^9^, we constructed a gene-gene-drug sub-network by compiling protein-protein interaction (PPI) network from InWeb^44^, drug-target associations from DrugBank and pharmacogenomic associations from PharmGKB. Hansen uses known drug-target and drug-pharmacogene (PGx) associations in human and a PPI network in order to build for each query protein a small subnetwork comprising of the protein, its immediate neighbors in the PPI network and their associated drugs. To rank possible PGx association for a query drug and gene it utilizes drug chemical similarity and indication similarity between the query drug and drugs associated with query gene neighbors in PPI. Subsequently, four features were constructed based on indication- or chemical- based drug similarity between the query drug and drugs associated with the query protein’s interactors by either PGx or drug-target associations. Finally, a logistic regression classifier was applied to these features to retrieve a score for each potential association. To facilitate the evaluation of the results we used our own similarity measures between drugs (based on chemical structure and ATC) and applied a Random Forest classifier, with the same set of positive and negative PGx associations.

We defined for each method a *valid* gold standard set as the set of PGx associations for which feature calculation is feasible; i.e. for Hansen’s method the valid set contains PGx associations between drugs annotated to either ATC category or chemical structure and proteins with at least one reported PPI. For our method, the valid set consists of associations between drugs annotated to both ATC category and chemical structure and proteins with sequence/domain similarity to yeast proteins. Overall, out of the 3075 PGx associations retrieved from PharmGKB, 1400 (45%) were valid for our method, while 389 (13%) associations were valid Hansen’s method. Out of these associations 140 PGx associations were valid for both methods (henceforth, the intersection set). We compared the performance of both methods, by training each method on the set of its valid associations minus the intersection set, and an equally sized set of random negative examples. The intersection set and an equally sized set of random negative associations were used as the test set.

To evaluate the contribution of HIP/HOP chemogenomic interactions to the PGx prediction task we applied our method on the same subset of PGx associations, omitting the chemogenomic interactions. For each query gene and query drug we constructed features based on known PGx in human, as the geometric mean of drug similarity and gene similarity between query pair and all known PGx associations as follow:


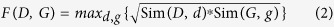


where D, G are the predicted drug and human gene, and *d, g* range over all known PGx between drugs and human genes in the training set. The sequence/domain gene similarities were calculated for all human genes as described above.

## Additional Information

**How to cite this article**: Silberberg, Y. *et al.* Utilizing yeast chemogenomic profiles for the prediction of pharmacogenomic associations in humans. *Sci. Rep.* **6**, 23703; doi: 10.1038/srep23703 (2016).

## Supplementary Material

Supplementary Dataset 1

Supplementary Dataset 2

Supplementary Dataset 3

Supplementary Dataset 4

Supplementary Dataset 5

## Figures and Tables

**Table 1:** A list of the eight features derived from each data source.

Feature formulation
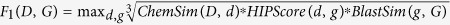
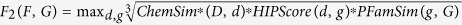
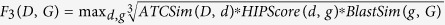
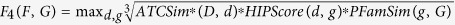
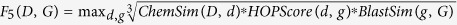
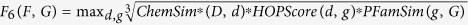
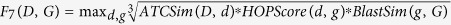
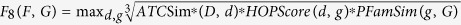

**Table 2:** Drugs predicted to have the highest numbers of associated PGx.

Drug name	Drug class
phenelzine	Antidepressive Agents | Monoamine Oxidase Inhibitors
trazodone	Antidepressive Agents, Second-Generation | Anti-Anxiety Agents | Serotonin Uptake Inhibitors
fluvoxamine	Antidepressive Agents, Second-Generation | Anti-Anxiety Agents | Serotonin Uptake Inhibitors
tacrine	Nootropic Agents | Cholinesterase Inhibitors | Parasympathomimetics
l-tryptophan	Antidepressive Agents, Second-Generation
amitriptyline	Antidepressive Agents, Tricyclic
amineptine	
paroxetine	Serotonin Uptake Inhibitors | Antidepressive Agents
iron	Trace Elements | Anti-anemic Agents | Supplements
tranylcypromine	Antidepressive Agents
mirtazapine	Histamine H1 Antagonists | Antidepressive Agents, Tricyclic | Adrenergic alpha-Antagonists
iproniazid	
nefazodone	Antidepressive Agents, Second-Generation
clomipramine	Serotonin Uptake Inhibitors | Antidepressive Agents, Tricyclic
duloxetine	
protriptyline	Adrenergic Uptake Inhibitors | Antidepressive Agents, Tricyclic
minaprine	Antidepressive Agents
vilazodone	Serotonin Uptake Inhibitors | Serotonin Receptor Agonists | Antidepressive Agents
citalopram	Antidepressive Agents, Second-Generation | Serotonin Uptake Inhibitors | Antidepressive Agents
sumatriptan	Vasoconstrictor Agents | Serotonin Antagonists
venlafaxine	Antidepressive Agents
reboxetine	
maprotiline	Antidepressive Agents, Second-Generation | Adrenergic Uptake Inhibitors | Antidepressive Agents
nortriptyline	Adrenergic Uptake Inhibitors | Antidepressive Agents, Tricyclic | Antidepressive Agents
amoxapine	Antidepressive Agents, Second-Generation | Serotonin Uptake Inhibitors | Adrenergic Uptake Inhibitors
doxepin	Adrenergic Uptake Inhibitors | Antidepressive Agents

Categories extracted from DrugBank and are ‘|’ delimited.

**Table 3:** Enriched categories in drugs with few PGx associations.

Drug category	Hyper geometric p-value	# drugs
Histamine H1 Antagonists	7.59E-09	22
Contrast Media	9.21E-09	11
Diagnostic Agents	4.98E-07	11
Anti-Inflammatory Agents, Non-Steroidal	1.26E-06	21
Bisphosphonates	8.06E-06	7
Muscle relaxant, Skeletal	1.12E-05	8
Anti-Inflammatory Agents	2.99E-05	24
Anti-Allergic Agents	5.56E-05	13
Histamine Antagonists	5.56E-05	13
Cyclooxygenase Inhibitors	2.75E-04	10
Histamine H1 Antagonists, Non-Sedating	1.19E-03	5
Antiresorptives	1.25E-03	4
Muscle Relaxants, Genitourinary	1.25E-03	4
Neuromuscular Nondepolarizing Agents	1.25E-03	4
Bronchodilator Agents	1.52E-03	10
Oxytocics	3.51E-03	5
Antihypocalcemic Agents	4.84E-03	7
Nicotinic Antagonists	5.33E-03	4
Anti-Incontinence Agents	6.69E-03	3
Cyclooxygenase 2 Inhibitors	6.69E-03	3
Bone Density Conservation Agents	7.35E-03	8
Muscle Relaxants, Central	8.51E-03	6
Indicators and Reagents	2.30E-02	3
Insecticides	2.30E-02	3
Neuromuscular Agents	2.71E-02	4
Carbonic Anhydrase Inhibitors	3.56E-02	2
Expectorants	3.56E-02	2
Hormone Replacement Agents	3.56E-02	2
Leukotriene Antagonists	3.56E-02	2
Muscle Relaxants, Respiratory	3.56E-02	2
Phosphodiesterase 5 Inhibitors	3.56E-02	2
Adrenergic alpha-1 Receptor Antagonists	4.63E-02	4
Anti-Asthmatic Agents	4.95E-02	3

**Table 4:** Cross validation and external evaluation on filtered set of drugs, removing drugs with high volume of predictions.

#Drugs removed	Maximal number of PGx predictions per drug	# positive examples in training set	Cross validation		AUC w.r.t. DrugBank associations		
			AUC	AUPR	Drug targets	Drug transporters	Drugs enzymes
26	600	1215	0.95	0.96	0.69	0.85	0.94
351	150	479	0.93	0.94	0.64	0.81	0.92

The filtered sets of drugs were evaluated by cross validation, reporting the AUC and AUPR scores. Next PGx predictions were obtained and compared to drug-gene associations extracted from DrugBank. Area under ROC curve for three gold-standard association types is reported.
